# A Choroidal Melanoma With Ciliary Body Involvement in a Young Female

**DOI:** 10.7759/cureus.56955

**Published:** 2024-03-26

**Authors:** Yen Sheong Lai, Ismail Shatriah, Yee Lin Lo, Koon Ling Koh, Jayaraman Kogilavaani

**Affiliations:** 1 Department of Ophthalmology and Visual Science, School of Medical Sciences, Universiti Sains Malaysia, Kubang Kerian, MYS; 2 Department of Ophthalmology, Hospital Raja Permaisuri Bainun, Ipoh, MYS

**Keywords:** metastasis, asian, female, young, ciliary body, choroidal melanoma

## Abstract

Choroidal melanoma with ciliary body involvement is rare, especially in young adults and Asians. Here, we report the case of a young, healthy Chinese woman who complained of decreased vision in the left eye for one week. Her ocular examination and imaging were suggestive of choroidal melanoma involving the ciliary body. The patient underwent enucleation of the left eye. Close monitoring was needed, as the involvement of the ciliary body in choroidal melanoma is associated with a high risk of metastasis.

## Introduction

Melanoma is a malignant tumor that originates from melanocytes at various body sites, with the ocular area being the second most common location after the skin [1]. The majority (85%) of ocular melanomas are uveal in origin [1,2], with the choroid being the most frequent site of origin. Ciliary body melanomas are rarely encountered and comprise 10% of all intraocular melanomas [3]. The common presentations of choroidal melanoma include flashes of light, floaters, visual field defects, and reduced vision [4]. Choroidal melanoma typically presents as an elevated, dome-shaped, gray-brown-colored lesion of the choroid [5].

Choroidal melanoma is more common in Caucasians (five to six cases per million) compared to Asians (0.2 cases per million). The mean presenting age is around the fourth to fifth decade of life in Asians, more than a decade earlier than in Caucasians [4]. Choroidal melanoma is found slightly more frequently in men, with a male-to-female ratio of 1.29 [2,4]. Here, we report on a rare presentation of choroidal melanoma with ciliary body involvement in a young Chinese woman.

## Case presentation

A healthy 23-year-old Chinese woman presented with sudden-onset painless blurred vision in her left eye for one week. She described her symptoms as generalized blurry vision associated with redness of her left eye and two episodes of photopsia. There were no floaters, no eye discharge, no loss of appetite or weight, no history of ocular trauma, and no family history of malignancy.

**Figure 1:**
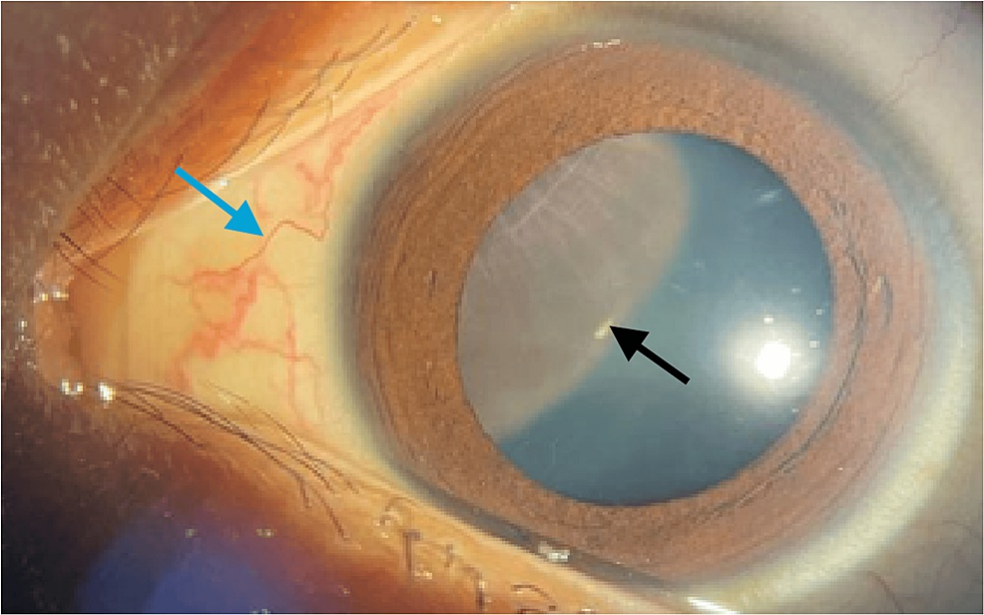
Anterior segment photo of the left eye showed a ciliary body mass (black arrow) with dilated conjunctival vessels (blue arrow)

**Figure 2:**
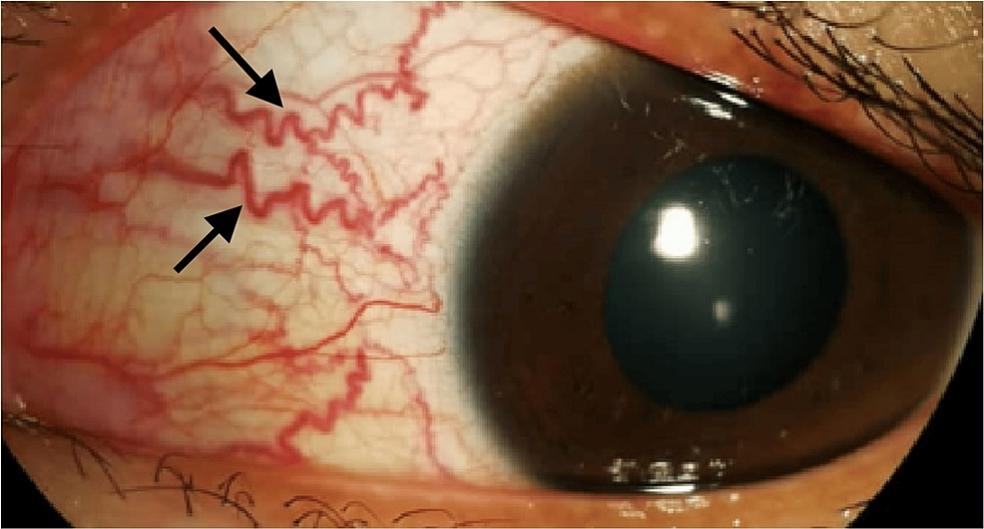
Anterior segment photo of the left eye showed sentinel vessels at the nasal conjunctiva (black arrows)

Ocular examination revealed a visual acuity of 6/9 in the right eye and 5/60 in the left eye, pinhole 6/18. A relative afferent pupillary defect was absent. The right eye finding was unremarkable. The anterior segment of the left eye revealed a large ciliary body mass located retrolental at the superonasal quadrant (Figure 1), causing striae at the superonasal of the posterior lens capsule associated with sentinel vessels at the nasal conjunctiva (Figure 2).

**Figure 3:**
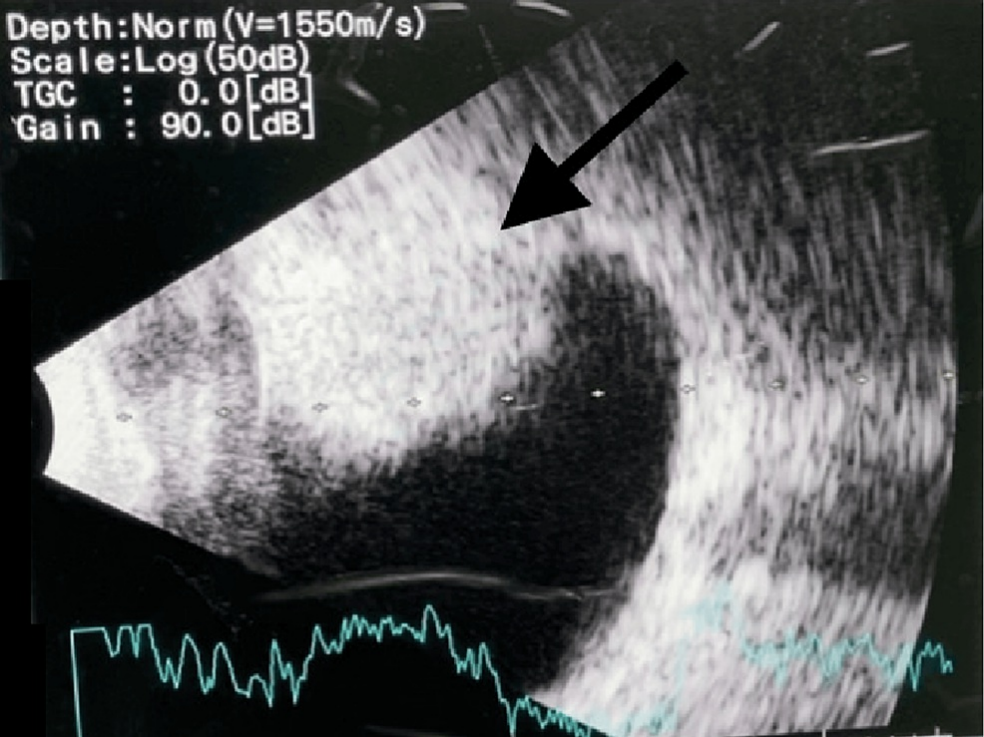
B-scan ultrasonography of the left eye at initial presentation, showing a well-circumscribed homogenous mass (black arrow)

Gonioscopy showed that all angles were open but unable to appreciate any mass. Fundus examination noted exudative retinal detachment at the inferonasal area of the periphery of the retina. A systemic examination was unremarkable. B-scan ultrasonography revealed a well-circumscribed homogenous mass at the superonasal aspect of the choroid (Figure 3).

**Figure 4:**
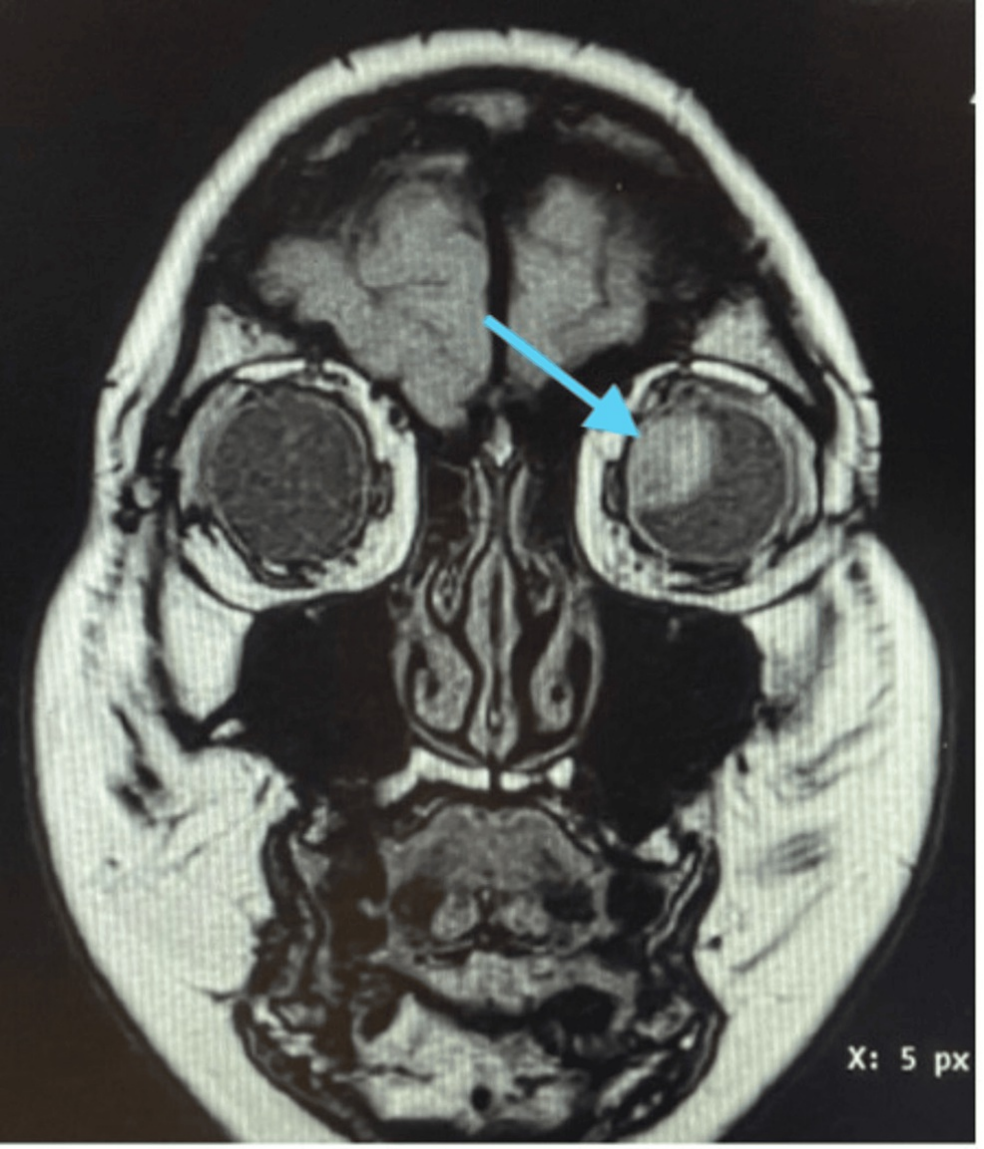
Coronal view T1 post-contrast MRI of the brain and orbits showed an enhancing lentiform-shaped intraocular lesion seen at the superomedial aspect of the left globe (blue arrow)

Blood investigations and tumor markers were normal, except for raised liver enzymes, which showed transaminitis. An ultrasound of the hepatobiliary system revealed hepatomegaly with hepatic steatosis, but neither a liver mass nor any lesions were noted. An MRI of the brain and orbits was suggestive of an enhancing lentiform-shaped intraocular lesion seen at the superomedial aspect of the left globe involving the sclera and ciliary body and possible infiltration at the attachment of the medial rectus (Figure 4).

**Figure 5:**
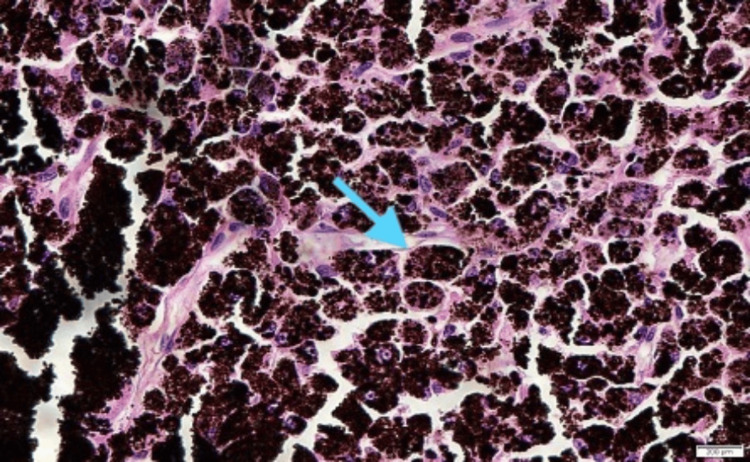
Histopathological examination (x400 magnification) showed epithelioid-appearing neoplastic cells with round to oval nuclei, prominent nucleoli, and abundant eosinophilic cytoplasm (blue arrow)

The patient was diagnosed with left eye choroidal melanoma involving the ciliary body and sclera. Left eye enucleation was done 20 days after presentation, and intraoperatively, no extrascleral extension was noted. Histopathological examination showed an epithelioid-type choroidal melanoma measuring 14 mm at the largest basal diameter, with ciliary body involvement (Figure 5).

Postoperatively, the patient was doing well. Her left eye socket wound was clean and intact, with no pigmented lesions. The patient remained under close follow-up every four months, with no evidence of recurrence or metastasis at one year.

## Discussion

Uveal melanoma is a malignancy of melanocytes that can be found in the uveal tract of the eye, which comprises the iris, ciliary body, and choroid. Choroidal melanoma is the most common of all subtypes of uveal melanoma. Risk factors for uveal melanoma include being Caucasian, elderly, and having ocular melanocytosis and oculodermal melanocytosis (nevus of Ota) [6].

Our patient was a Chinese woman in her early 20s with no comorbidities or predisposing risk factors. Thus, it was unusual for her to have choroidal melanoma. To date, there have been five published cases of uveal melanoma affecting people in their 20s, and most were Caucasian, irrespective of gender. All five cases had uveal melanoma in different clinical presentations, but none involved the ciliary body. Our patient’s Chinese ethnicity and presentation of a choroidal mass involving the ciliary body made her different from the published case reports. Ciliary body involvement is associated with a high risk of metastasis owing to the rich blood supply [3]. The diagnosis of uveal melanoma is mainly clinical; thus, a high level of clinical suspicion is needed. Table 1 summarizes the published reports on uveal melanoma diagnosed in patients in their 20s [7-11], including our patient.

**Table 1:** Summary of published case reports of uveal melanoma in patients in their 20s

Authors/year	Age/gender/race or country	Risk factor	Presentation	Examination	Treatment	Outcome
Pomeranz et al. (1981) [7]	29/female/White	Ocular melanocytosis	Blurred vision	Two separate choroidal masses with shallow retinal detachment	Enucleation	Not available
de A Silva et al. (2011) [8]	28/female/Brazilian	History of right eye enucleation 10 years ago for choroidal melanoma	Intense headache	The left frontal lobe, single metastatic melanoma	Whole-brain radiation therapy	No recurrence for 24 months post-radiation or death from pulmonary embolism
Nair et al. (2014) [9]	24/male/Asian Indian	Nil	Sudden loss of vision, preceded by painful, progressive proptosis of the right eye (orbital cellulitis)	A B-scan showed a large dome-shaped mass filling the posterior segment	Three days of intravenous dexamethasone, intravenous antibiotics, topical steroids, and cycloplegic eye drops, followed by enucleation	There is no evidence of systemic metastasis
Gupta et al. (2016) [10]	26/male/United States	Nil	Headache and a red, painful eye with nausea and vomiting	Neovascular glaucoma, necrotizing scleritis, and exudative retinal detachment	Enucleation	Not available
Hackett et al. (2018) [11]	21/female/United States	History of right optic nerve melanocytoma	Transient right-sided visual field deficits, floaters, unilateral headache, and photopsia	Dark-pigmented vascular mass obscuring the optic nerve with no extension to the surrounding orbit	Referred to a retina specialist for further management	Not available
Our patient (2024)	23/female/Asian (Chinese)	Nil	Blurred vision and eye redness	Choroidal mass involving the ciliary body	Enucleation	No systemic metastasis

Computed tomography and MRI are important for identifying tumor extension and ruling out metastases, if present. The main treatment options include enucleation, brachytherapy, transpupillary thermotherapy, and orbital exenteration for tumors with orbital invasion [12]. In our case, enucleation was performed because there was no extrascleral extension.

A better prognosis for choroidal melanoma is observed in Asian populations due to the younger age of onset, with a five-year survival rate of more than 70% [6]. Large basal tumor diameters, ciliary body involvement, a non-spindle cell type, and extrascleral extension are worse prognostic indicators [6,13]. Tumor sizes are classified into small (11 mm diameter), medium (11-15 mm diameter), or large (15 mm diameter), with a 10-year survival rate of 81% for small melanomas, 60% for medium melanomas, and 35% for large melanomas [13]. Our patient had a medium-sized melanoma. Therefore, long-term, regular systemic follow-up is necessary, as the prognosis is poor once metastases have developed.

## Conclusions

Uveal melanoma is a disease that usually affects elderly Caucasians. However, the possibility of its occurrence in younger patients of other races should not be overlooked. Misdiagnosis is common when patients are not properly evaluated and investigated, leading to delays in treatment and, consequently, the development of metastatic disease.
